# Phosphatidylethanolamine-phosphatidylserine binding synergy of seven coagulation factors revealed using Nanodisc arrays on silicon photonic sensors

**DOI:** 10.1038/s41598-020-73647-3

**Published:** 2020-10-15

**Authors:** Sara M. Medfisch, Ellen M. Muehl, James H. Morrissey, Ryan C. Bailey

**Affiliations:** 1 Department of Chemistry, University of Michigan, 930 N. University Avenue, Ann Arbor, MI 48109 USA; 2 Department of Biological Chemistry, University of Michigan, 1150 W. Medical Center Drive, Ann Arbor, MI 48109 USA

**Keywords:** Lipids, Biochemistry, Analytical biochemistry, Biophysical methods

## Abstract

Blood coagulation is regulated through protein–protein and protein–lipid interactions that occur at the sub-endothelium following vascular damage. Soluble clotting proteins bind to membrane components in a phosphatidylserine (PS) dependent manner to assemble multi-protein complexes that regulate clot formation; however, PS is of limited abundance physiologically. In this manuscript, we investigate synergy between PS and phosphatidylethanolamine (PE)—a lipid of much higher abundance naturally. Using a label-free, silicon photonic technology, we constructed arrays of Nanodiscs having variable lipid composition and probed the binding interactions of seven different clotting factors with GLA domains that have never been studied in tandem experiments before. The factors studied were prothrombin, activated factor VII, factor IX, factor X, activated protein C, protein S, and protein Z. Equilibrium dissociation constants (*K*_*d*_) for each coagulation factor binding to Nanodiscs with unique compositions of PE and PS were determined. While all factors showed greater binding affinities in the presence of PS and PE, the most dramatic improvements in binding were observed when PS quantities were lowest. This demonstrates that synergy is effective in promoting coagulation factor binding under physiological lipid compositions, as opposed to the artificially high PS content probed in most in vitro activity studies.

## Introduction

The phospholipid content of the cell membrane is a key regulator of the blood coagulation cascade^1,2^. Exposure of tissue factor and the negatively charged lipid, phosphatidylserine (PS), via cell membrane damage is one of the initiating factors of the cascade^3,4^. The majority of steps in the clotting cascade occur at the membrane surface and involve multiple PS lipid-binding proteins^4^. The most common PS-binding domain found in both pro- and anti-coagulant factors is the γ-carboxyglutamic acid-rich GLA domain located at the N-terminus of these proteins [Procoagulants: factor VII (fVII); factor IX (fIX); factor X (fX); and prothrombin (PT). Anticoagulants: activated protein C (aPC); protein S (PrS); and protein Z (PrZ).] The GLA domain reversibly binds to PS lipids in a calcium dependent manner^4^. While the structures of the GLA domains are highly conserved, they display membrane binding affinities that vary by more than two orders of magnitude^2,4^, which suggests differences in the lipid binding preferences of each.

Even though PS lipids are necessary for optimal activity of many clotting factors, they only constitute ~ 12% of the phospholipid content of the cell membrane^5^, which is much lower than what is required for optimal procoagulant activity of activated fVII (fVIIa) in vitro (~ 30% PS in liposomes)^1^. Previous studies have shown that lipid composition influences the binding and activity of GLA domain-containing clotting proteins. For example, even though GLA domains of clotting proteins are structurally and compositionally homologous, aPC and fVIIa bind phosphatidic acid (PA) lipids much more tightly than PS lipid^2^. Maximal rates of activation of fX by fVIIa bound to tissue factor^6–8^, of activation of prothrombin by fXa bound to factor Va^9^, and of inactivation of factor Va by aPC^10^, have been reported to require much less PS when membranes also contain phosphatidylethanolamine (PE). This phenomenon has been termed PE–PS synergy. The “Anything But Choline” (ABC) hypothesis explains PE–PS synergy by stating that almost any phospholipid, other than those with choline head groups, can work cooperatively with PS lipids to greatly reduce the amount of PS required for binding and activation of clotting proteins^8^. PE comprises ~ 25% of the phospholipid content of the plasma membrane and is normally sequestered, along with PS, in the inner leaflet of the bilayer^5^. Cell membrane damage exposes both PE and PS to support of blood coagulation reactions, showing physiological relevance for PE–PS synergy.

Since most of the previous work on PE–PS synergy focused on enzyme activity assays using membrane-bound enzyme, cofactors and substrates, it is not clear which of the proteins within these multi-protein complexes actually exhibited enhanced membrane binding in the presence of PE. This is especially important since some of the proteins in these complexes (e.g., factors Va and VIIIa), bind to PS in membrane surfaces via discoidin-type C2 domains, not GLA domains^4^. In fact, to date, the only GLA domain-containing protein whose binding to membrane surfaces has been directly shown to be enhanced by PE is fX, raising the question of how generally PE–PS synergy promotes the binding of GLA domain-containing proteins.

**Figure 1:**
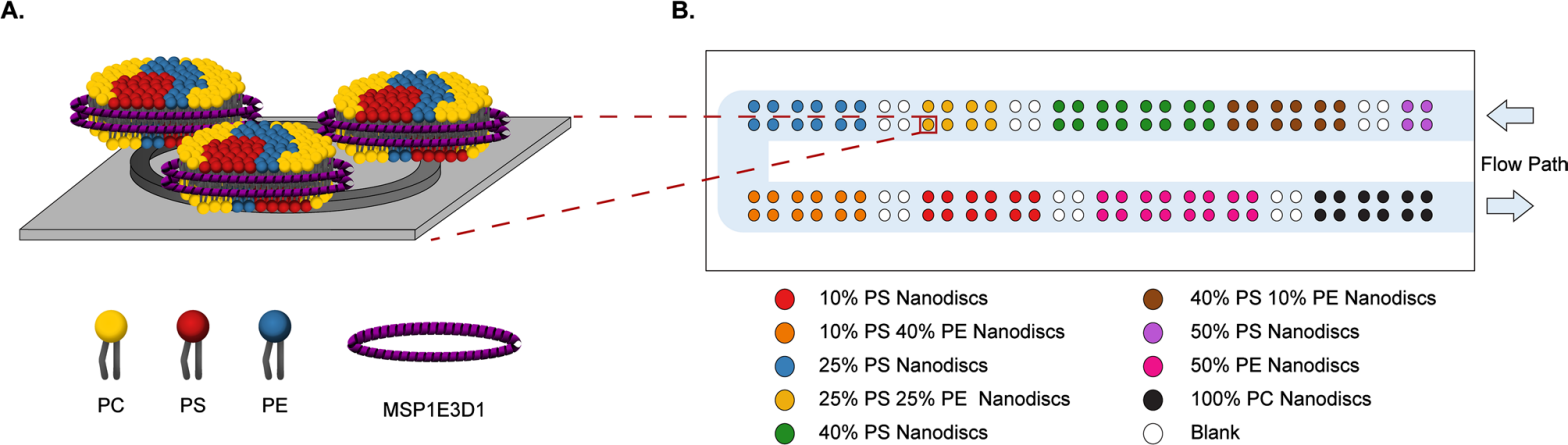
(**A**) Schematic of Nanodisc physisorption to a microring resonator. The MSP (purple) stabilizes the lipids (PC: yellow; PS: red; and PE: blue) to form the Nanodisc bilayer mimetic. *Note* The schematic is not to scale. The microring resonator is 30 µm in diameter while the Nanodiscs are about 13 nm in diameter and 5 nm in thickness. (**B**) Spotting layout of the sensor chip for protein titrations. The flow path for experiments is shown in light blue. Figure created using Adobe Illustrator CC2020 (https://www.adobe.com/products/illustrator.html).

The aim of this study was to comprehensively and quantitatively evaluate the degree to which PE–PS lipid synergy enhances membrane binding of all seven GLA domain-containing proteins of the clotting cascade in tandem experiments. As a model membrane system, Nanodiscs were constructed to present well-defined, variable lipid compositions. Nanodiscs are small lipid bilayer discs held together by two membrane scaffold proteins (MSPs) that offer a high degree of control over lipid composition^11,12^. Nanodiscs have proven to be a useful tool in the study of membrane-lipid interactions of the blood coagulation cascade^1,2,8^. Various Nanodisc were spatially arrayed onto a label-free and highly multiplexable silicon photonic detection platform to allow in series determination of binding constants (Fig. 1). Recently, we demonstrated the ability to utilize this combination of Nanodiscs and microring resonator sensors for high-throughput interrogation of protein binding to model membrane surfaces^13,14^, including coagulation factors, PT, fX, fVIIa, and aPC. This work demonstrated that binding interactions can be rapidly probed simultaneously with reduced time and reagent consumption compared to conventional methods. Herein, we further extend this technology to probe the binding interactions of all seven GLA domain-containing coagulation factors to Nanodiscs presenting defined ratios of PS and PE. These experiments were performed in series on a single array of Nanodiscs to eliminate environmental changes day-to-day that can result in variation in array formation. Thus, providing an optimal surface for direct comparison of binding improvements due to lipid composition.

More specifically, we determined *K*_*d*_ values for fVIIa, fIX, fX, PT, aPC, PrS, and PrZ binding to Nanodiscs at eight distinct lipid compositions. We directly compared *K*_*d*_ values of binding to Nanodiscs of 10, 25, 40 and 50% PS to Nanodiscs containing 10% PS/40% PE, 25% PS/25% PE, and 40% PS/10% PE, or 50% PE. The balance in all Nanodiscs was made up with non-interacting phosphatidylcholine (PC) lipids. Utilizing silicon photonic microring resonators as our sensing platform, we have shown that sequential titrations can be performed on a Nanodisc array surface^14^. Thus, experiments with each clotting factor were performed sequentially on the same Nanodisc array surface to eliminate day-to-day variation observed with other experimental setups.

We found that PE had a significant effect on *K*_*d*_*, *driving much tighter binding of all factors to the model membrane surface. Using these calculated *K*_*d*_ values, we quantitated the fold-change in binding due to the addition of PE to PS. Interestingly, the magnitude of binding improvement was greatest at the lowest PS contents, consistent with physiological lipid abundances. This synergistic improvement in binding was more striking for the clotting proteins that showed poorest binding to PS alone which have not been shown to observe this synergy—fVIIa, PrZ, and aPC. These proteins yield very high (or unmeasurable) *K*_*d*_ values (poor binding) at near physiological PS content and therefore PE–PS synergy appears essential for regulation of coagulation. This work offers a comprehensive and quantitative view of the synergistic influence of PE on binding of clotting cascade proteins across all seven GLA domain-containing clotting proteins and is a high-throughput complement to activity-based studies of their pro- and anticoagulant activities modulated by protein–lipid interactions.

## Materials and methods

### Chemicals and reagents

Lipids presenting phosphocholine (POPC; 1-palmitoyl-2-oleoyl-sn-glycero-3-phosphocholine), phosphoserine (POPS; 1-palmitoyl-2-oleoyl-sn-glycero-2-phosphoserine), and phosphoethanolamine (DOPE; 1,2-dioleoyl-sn-glycero-3-phosphoethanolamine) head groups were purchased from Avanti Polar Lipids (Alabaster, AL). MSP1E3D1 was expressed in *E. coli* and purified as described previously^12^. Human fVIIa, fX, PT, aPC, and PrZ were purchased from Enzyme Research Laboratories Inc. (South Bend, IN). Human fIX and PrS were purchased from Haematologic Technologies (Essex Junction, VT). Amberlite XAD-2 hydro-phobic beads and all other chemicals were purchased from Sigma Aldrich (St. Louis, MO) and used as received unless otherwise noted. Buffers were prepared with 18.2 MΩ water and sterile filtered prior to use.

### Solution preparation

Nanodisc solutions were prepared in a TBS buffer (20 mM Tris–HCl, 100 mM NaCl, and 0.01% (w/v) NaN_3_; pH 7.4). PT, fIX, and fX solutions were prepared in a HEPES buffer (10 mM HEPES, 150 mM NaCl, 50 μM EDTA, 2.5 mM CaCl_2_, 0.1% (w/v) PEG 8000; pH 7.4). Solutions of fVIIa, aPC, PrS, and PrZ were prepared in HEPES buffer with 0.2% (w/v) BSA. The HEPES rinse buffer (HEPES(−)) for surface regeneration was made without CaCl_2_.

### Nanodisc preparation and purification

Nanodisc preparation and purification has been described in detail previously^11,12,15^. Briefly, lipids solubilized in chloroform were measured into test tubes and dried under nitrogen. For Nanodiscs containing mixtures of POPS, DOPE, and/or POPC, the lipids were mixed at defined ratios prior to drying. After drying, lipids were placed in a desiccator, under vacuum overnight. Once completely dry, lipids were dissolved in TBS buffer with 100 mM deoxycholate to give a final molar ratio of 2:1 dexoycholate:phospholipids. Dissolved lipids were then combined with MSP1E3D1 in TBS to give a final molar ratio of 135:1 phospholipid:MSP. The solution of MSP and lipids was actively mixed at 4 °C for approximately 1 h. Amberlite XAD-2 hydrophobic beads were then added to the MSP/lipid solution with the amount of bead solution being half the total of the MSP/lipid solution volume. This combined solution was then mixed for approximately 1.5 h at 4 °C. Amberlite XAD-2 hydrophobic beads were then removed by filtering through a 0.22 μm syringe filter. Nanodiscs were then purified using size exclusion chromatography using a Superdex 200 column (GE).

### Silicon photonic microring resonators

The Maverick M1 optical scanning instrumentation and microring resonator sensor chips were purchased from Genalyte, Inc. (San Diego, CA). The operation of the instrument has been previously described^16–20^. The sensor chips were each 4 mm × 6 mm and contained 128, 30-μm diameter active sensor microrings arranged in clusters of four, plus four temperature control microrings and two dedicated to detecting leaks from the microfluidic gasket positioned atop the sensor chip during microring detection experiments.

### Sensor chip array functionalization

Prior to use, sensor chips were placed in a vial of acetone for 2 min with gentle agitation. Sensor chips were then transferred to a vial of isopropanol for 2 min. After the sensor chips had been dried with N_2_, between 0.1 and 0.2 μL of each type of Nanodisc were spotted at 0.5 μM. A spotting map showing the arrangement of the Nanodisc solutions on the sensor substrate is shown in Fig. S1. After spotting, chips were stored in a humidity chamber at 4 °C for at least 1 h before use.

### Protein binding titrations

Laser-cut Mylar gaskets to direct fluid flow across the chip were aligned onto the functionalized sensor chips, assembled into a Teflon cartridge, and loaded into the sensor scanner instrument. A 2% solution of BSA in HEPES (−) buffer was first flowed across the chip surface at 10 μL/min to prevent the non-specific binding of proteins. For *K*_*d*_ determination titrations, the proteins were flowed across the chip in increasing concentrations at 10 μL/min and the response allowed to approach steady state before the next solution injection. Following each titration, all of the coagulation factors were released from the surface by flowing HEPES (−) buffer solution. The titration was then performed for the next protein.

### Data analysis

Data analysis was performed using custom R scripts in RStudio. Sensor traces were corrected for temperature fluctuations and any residual non-specific binding by subtraction of response of 100% PC Nanodiscs. The maximum shift for each protein concentration was calculated by subtracting the relative shift (Δpm) at the equilibration point for HEPES buffer (at 10 min) from the relative shift (Δpm) at equilibration for each protein concentration (the curve plateau). This maximum shift is plotted versus the protein concentration for fitting to the single-site ligand binding equation in order to determine *K*_*d*_ values:

1 $$\mathrm{\Delta pm}={B}_{max}\left(\frac{X}{{K}_{d}+X}\right)$$

where *X* is the concentration of protein, *B*_*max*_ is the maximum binding shift, and *K*_*d*_ is the equilibrium dissociation constant. Fold-changes were calculated as ratios of *K*_*d*_ values without PE to those with PE, at each identical PS content. Standard errors in the ratios were determined based on Fieller’s Theorem with independent values^21^.

## Results and discussion

### Sensor array layout

PS lipids are necessary for binding of GLA domain-containing clotting proteins to membranes, yet PS constitutes only ~ 12% of the phospholipid content of the plasma membrane^5^. Due to the fact that PE is one of the most abundant phospholipids in the membrane (~ 25%), it has the potential to play a significant supporting role to PS lipids in clotting protein binding and activation, especially for those proteins with low PS binding affinity. In order to assess the influence of PE lipids on binding, the equilibrium dissociation constants, *K*_*d*_ values, of fVIIa, fIX, fX, PT, aPC, PrS, and PrZ, were determined for eight different lipid compositions using the same Nanodisc microarray to prevent experiment to experiment variation. The lipid compositions included four binary mixtures of PS and PC (10, 25, 40, and 50% PS with the balance PC), one binary mixture of PE and PC (50% PE and 50% PC), three ternary mixtures of PS, PE, and PC (10%/40%/50%, 25%/25%/50%, and 40%/10%/50% PS/PE/PC, respectively), and 100% PC as an off-target control. Figure 1B shows the layout of the spotted Nanodisc array sensor chip.

### Effect of lipid composition on ***K***_***d***_ values for membrane binding of all 7 Gla-domain containing clotting proteins

**Figure 2:**
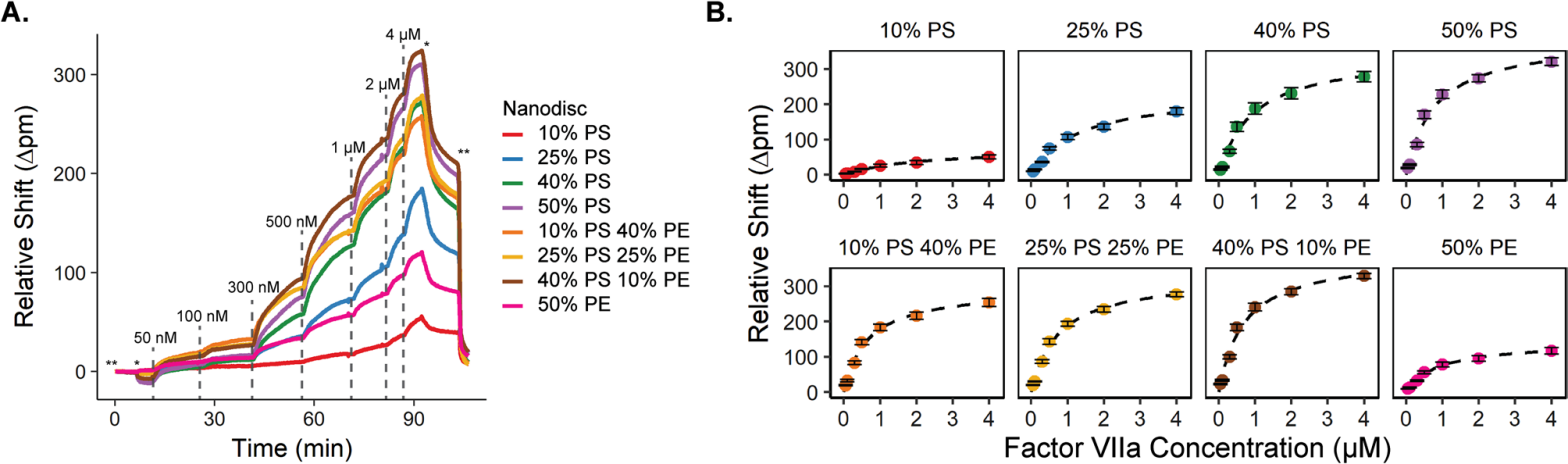
(**A**) Binding titration of activated factor VII (fVIIa) to Nanodiscs of the eight different lipid compositions 10% PS (red), 25% PS (blue), 40% PS (green), 50% PS (purple), 10% PS/40% PE (orange), 25% PS/25% PE (yellow), 40% PS/10% PS (brown), and 50% PE (pink), with the lipid balance of PC lipids. In all cases, background binding to 100% PC Nanodiscs was subtracted to correct for non-specific interactions. *Dashed lines* indicate time points where a new concentration of fVIIa was added (ranging from 50 to 4000 nM). The *marks the transition to HEPES buffer, and **marks the transition to HEPES(−) to initiate surface regeneration. (**B**) Relative resonance wavelength shift as a function of fVIIa concentration for each Nanodisc type. Equation (1) was used for fitting. Error bars represent standard deviation of at least n = 4 microrings. Plots generated in RStudio 1.1.453 and R 3.5.1 (https://www.R-project.org/) using the ‘ggplot2’ package.

Binding titrations for each protein were sequentially performed to determine the *K*_*d*_ values across each of the eight different lipid compositions. As an example, the binding titration of fVIIa, monitored in real time via a shift in the resonant wavelength of the microring sensors on the Nanodisc-arrayed sensor chip is presented in Fig. 2A. The concentration of fVIIa was increased in a stepwise fashion from 50 to 4000 nM. The titration shows preferential binding of fVIIa to PS-containing bilayers when PE lipids were also present in the Nanodiscs. At the end of the titration, HEPES (−) was flowed across the surface to regenerate a clean Nanodisc array for the next protein titration in the sequence. The 100% PC lipid Nanodiscs were used as a control due to the lack of specific binding of GLA domains to PC-Nanodiscs. For data analysis, the minimal binding of fVIIa to the PC-Nanodisc functionalized rings was subtracted to correct for any nonspecific binding. The maximum shift at each step of the titration versus concentration were then fit with a single-site binding model (Fig. 2B). The titrations experiments that were used in *K*_*d*_ determination for the other six clotting factors are shown in Figs. S1–S6.

**Figure 3:**
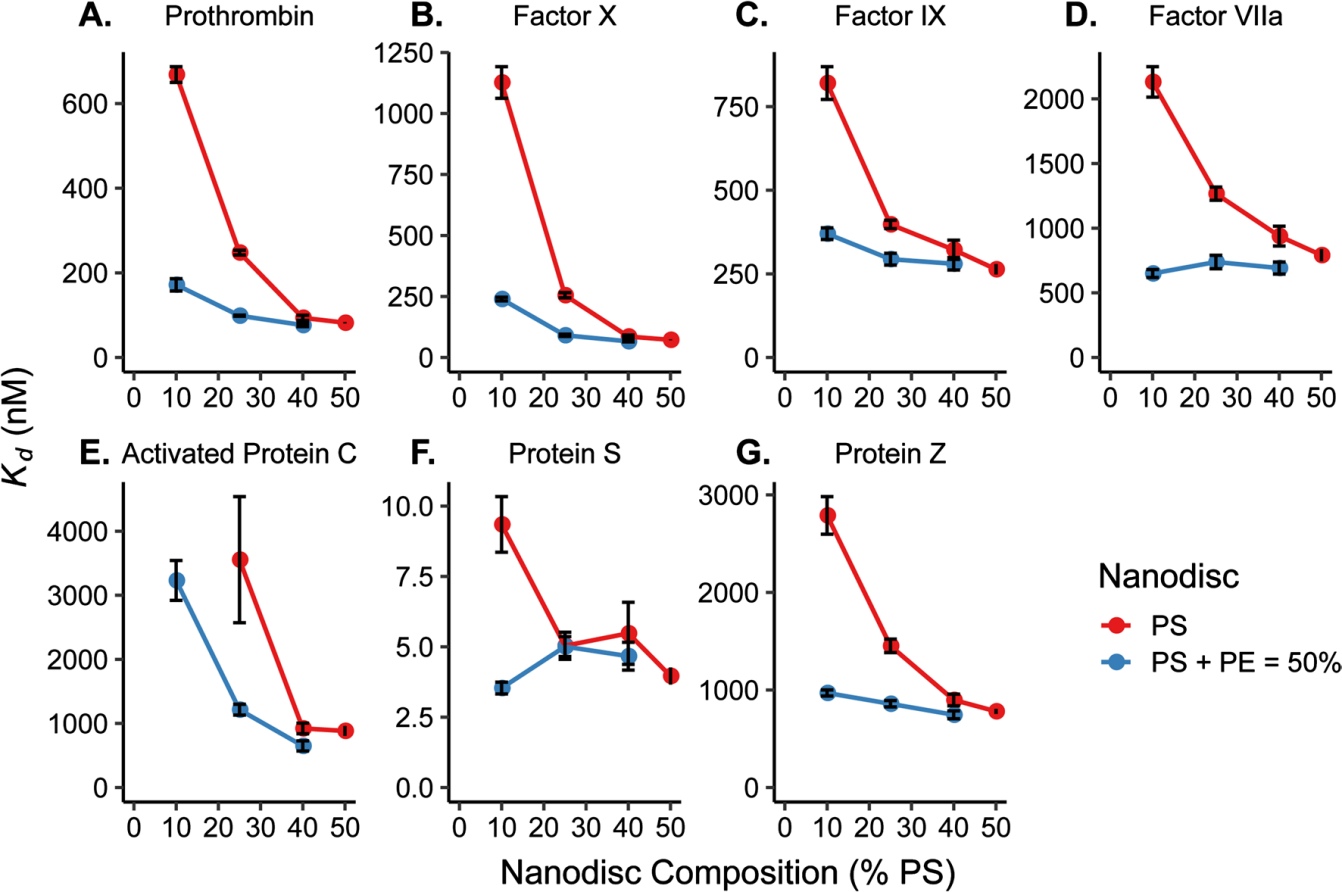
Comparison of *K*_*d*_ values from binding of (**A**) PT, (**B**) fX, (**C**) fIX, (**D**) fVIIa, (**E**) aPC, (**F**) PrS, and (**G**) PrZ to Nanodiscs with (blue) and without (red) PE lipids. *K*_*d*_ values are plotted as a function of percent PS where the PS + PE lipid composition is equal to 50% for the PE lipid containing Nanodiscs. Each was determined by plotting relative shift of binding vs. concentration of protein and fitting to Eq. (1). Error bars represent the standard deviation from at least n = 4 microrings. *K*_*d*_ values for 50% PE/50% PC are not shown in the graphs due to the large error from the poor binding to this lipid environment by each clotting factor. # aPC binding to 10% PS without PE was too weak to calculate *K*_*d*_. Plots generated in RStudio 1.1.453 and R 3.5.1 (https://www.R-project.org/) using the ‘ggplot2’ package.

The dissociation constant, *K*_*d*_, for a binding event is defined as the concentration at which half of the binding sights in an interaction are occupied. Therefore, the a lower *K*_*d*_ value for an interaction, the less analyte is needed to occupy these sites and the higher affinity the analyte has for the binding interaction. Here, *K*_*d*_ is measured directly using single-site ligand binding equation, but this value can also be calculated from the on- and off-rates (*k*_*on*_ and *k*_*off*_) for a binding and dissociation event. Using this method, the ratio of the *k*_*off*_/*k*_*on*_ is used to determine *K*_*d*_. The rate constant definition still shows increased binding affinity with decreasing *K*_*d*_ since this would mathematically translate to a slower off-rate or a faster on-rate which correlate to a favored binding state. Conversely, an increase in *K*_*d*_ between interactions show that the binding even is less favorable due to the adjusted conditions. Thus, observing interactions between different lipid environments and proteins, a low *K*_*d*_ can be used to determine the lipids that the protein has a higher affinity for binding. Figure 3 shows a comparison of the *K*_*d*_ values obtained for each clotting protein interacting with all tested lipid environments. Note that the *x*-axis in the figure indicates the percentage of PS in each Nanodisc. The blue lines indicate trends in *K*_*d*_ for Nanodiscs containing PE and red lines are for Nanodiscs without PE. Table S1 contains the numerical values for *K*_*d*_ values at each Nanodisc composition. For all seven clotting proteins, the *K*_*d*_ values for binding to Nanodiscs containing PE and PS are lower (stronger binding) compared to those with PS but without PE—even at identical total amounts of PS—which is indicative of PE–PS synergy.

### Quantifying the effect of lipid synergy membrane binding

The significance of PE lipids’ influence on the binding of the clotting factors to the lipid bilayer can be most clearly seen in the cases of fVIIa and aPC, both of which bind relatively poorly to PS/PC bilayers. aPC and fVIIa have an even greater binding affinity towards PA lipids^2^, but PA lipids only constitute 1–2% of the plasma membrane^22^. Figure 3D,E show the *K*_*d*_ values for fVIIa and aPC respectively. aPC binding to Nanodiscs with 10% PS/90% PC was unmeasurably weak (Fig. S4B), but upon addition of 40% PE lipids, binding was clearly observed. In fact, the addition of PE resulted in stronger fVIIa binding than for the 25% PS/75% PC Nanodiscs. *K*_*d*_ values obtained for fVIIa binding to any ratio of PS to PE lipids were lower than that for 50% PS Nanodiscs. PrS (Fig. 3F) showed extremely tight PS lipid binding affinity for all PS content, with the most dramatic synergy observed at low PS content (10% PS with 40% PE). Taken altogether, it is clear that PS is required for the binding of clotting factors to membrane surfaces; however, the addition of PE, which is in much higher natural abundance compared with PS and PA, can dramatically increase the binding affinities through synergistic effects. Further measurements that examine higher order lipid mixtures using this high-density sensor array technology could give additional insights into more complex synergistic binding interactions that help regulate coagulation in vitro.

**Figure 4:**
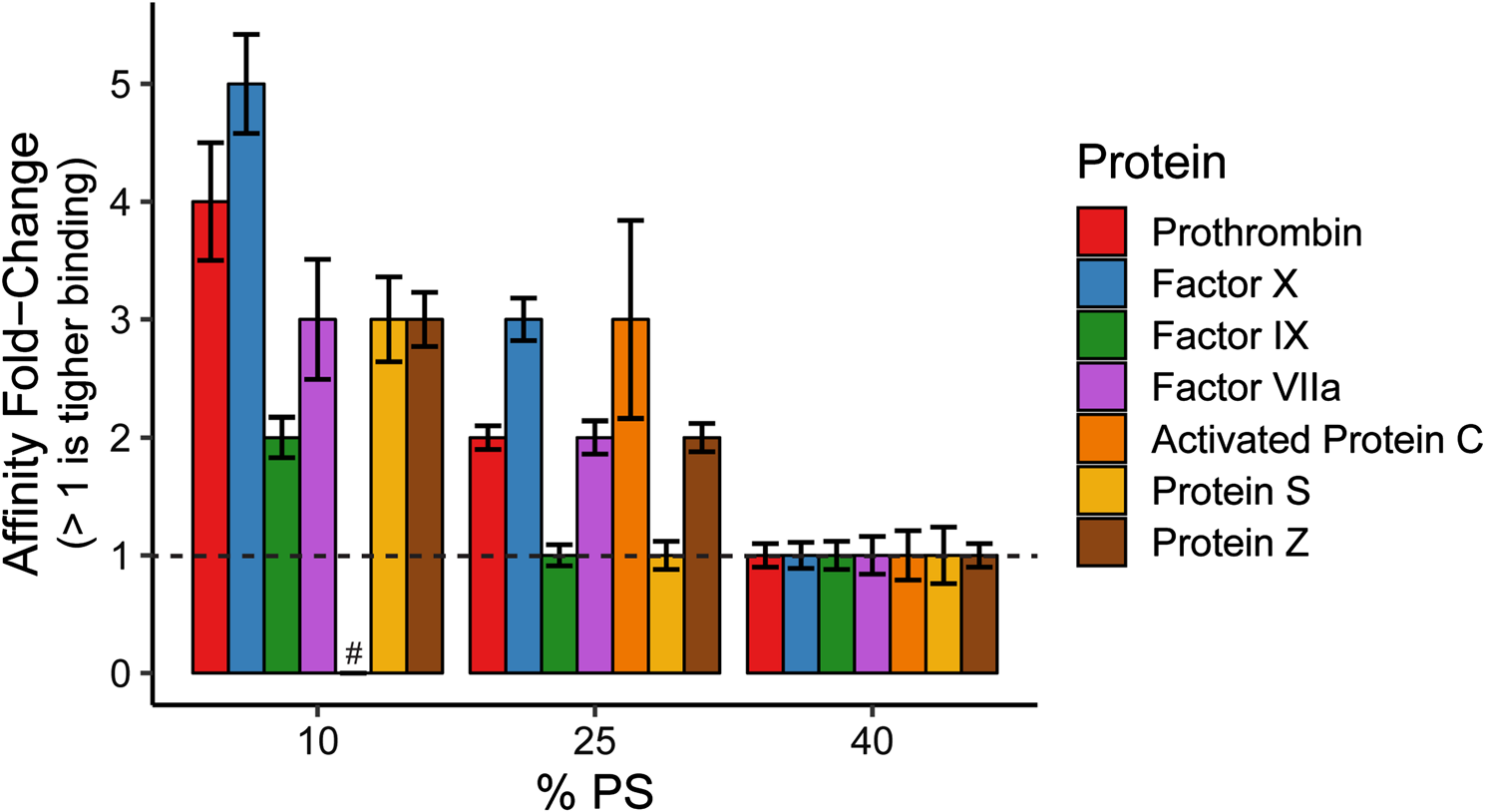
Affinity increase (*K*_*d*_ decrease) due to PE–PS synergy. These values were calculated by taking the ratio of *K*_*d*_ values without PE to those with PE. The *dashed line* represents no change in *K*_*d*_ with the addition of PE. Error bars represent standard error in calculating the ratio of these two values^21^. # Fold-change unable to be calculated since aPC binding to 10% PS without PE was too weak to measure. Plot generated in RStudio 1.1.453 and R 3.5.1 (https://www.R-project.org/) using the ‘ggplot2’ package.

Overall, the data collected from the binding titrations of the seven GLA domain-containing clotting proteins demonstrated that inclusion of PE lipids reduced the concentration of PS lipids needed for the strongest binding affinities. This work complements previous studies^6–10^ that examined the synergistic effect of PE lipids to support activity of clotting proteins. The correlation of *K*_*d*_ values with the activity suggests that PE synergizes with PS to improve the binding and potentially enhance the activity of all GLA domain-containing clotting proteins under conditions of low PS content. While PE affected the binding of all the proteins within this study, the magnitude of PE–PS synergy varied. To more clearly show these differences, Fig. 4 shows the relative enhancement of binding affinity via the inclusion of PE for each given PS concentration. This also nicely highlights that the synergy is most effective at low PS compositions, which is important as these are the most physiologically relevant lipid compositions in the array.

## Conclusion

Here, we utilized a high-throughput approach to rapidly screen the binding of all seven GLA domain-containing coagulation factors (fVIIa, fIX, fX, PT, aPC, PrS, and PrZ) interacting with Nanodiscs with nine different combinations of PS, PE, and PC lipids. This comprehensive study affirms previous observations of PE–PS synergy for some of these proteins from activity-based assays, but also reveals new protein-lipid synergies. Importantly, this platform allows for internally controlled measurements since the same sensor array can be regenerated and the measurements are performed under identical conditions in a single day. Furthermore, different magnitudes of synergy were observed for different clotting proteins which have never been compared before. The enhancements in binding affinity were more pronounced at lower PS contents, which are most similar to true in vivo lipid compositions. This is in contrast to most in vitro experiments that use extremely high amounts of PS in both binding and activity assays. These observations of PE–PS synergy with all GLA domain-containing blood coagulation factors have been performed on model membrane mimetics in vitro; thus, the application shows the ability to characterize biological interactions but is not able to accurately depict what is occurring in the endothelium. However, this quantitative look at lipid synergy with PS and PE provides biological understanding to the lipid binding affinity of the GLA domain and different the binding affinity differences between GLA domain-containing proteins. In the future, more complex, multi-component mixtures can be explored to reveal more nuanced binding synergies. Furthermore, other PS-binding proteins such as Matrix GLA protein, Growth-Arrest-specific protein 6, factor VII and factor VIII could be investigated to see if these non-clotting factor proteins exhibit similar PE–PS binding synergies.

## Supplementary information

Supplementary Information.

## Abbreviations

PS: Phosphatidylserine
PE: Phosphatidylethanolamine
PA: Phosphatidic acid
PC: Phosphatidylcholine
PT: Prothrombin
fX: Factor X
fIX: Factor IX
fVIIa: Activated factor VII
aPC: Activated protein C
PrS: Protein S
PrZ: Protein Z
