# Supplementary material for: Phosphatidylethanolamine-phosphatidylserine binding synergy of seven coagulation factors revealed using Nanodisc arrays on silicon photonic sensors
Source: Sci Rep. 2020 Oct 15;10:17407. doi: 10.1038/s41598-020-73647-3 (PMC7562748; doi:10.1038/s41598-020-73647-3)
Supplement: Supplementary file 1 — Supplementary Information. [file 41598_2020_73647_MOESM1_ESM.pdf]

Phosphatidylethanolamine-Phosphatidylserine Binding Synergy of Seven Coagulation Factors Revealed Using Nanodisc Arrays on Silicon Photonic Sensors

Sara M. Medfisch,[1,3] Ellen M. Muehl,[1,3] James H. Morrissey,[2] and Ryan C. Bailey[1,*]

[1]Department of Chemistry, University of Michigan, 930 N. University Avenue, Ann Arbor, Michigan 48109, United States; [2]Department of Biological Chemistry, University of Michigan, 1150 W. Medical Center Drive, Ann Arbor, Michigan 48109, United States; [3]These authors contributed equally: Sara M. Medfisch and Ellen M. Muehl

Figure S1: Prothrombin (PT) K_d determination S2

Figure S2: Factor X (fX) K_d determination S3

Figure S2: Factor IX (fIX) K_d determination S4

Figure S4: Activated protein C (aPC) K_d determination S5

Figure S5: Protein S (PrS) K_d determination S6

Figure S6: Protein Z (PrZ) K_d determination S7

Table S1: K_d values of PT, fX, fIX, fVIIa, aPC, PrS, and PrZ S8

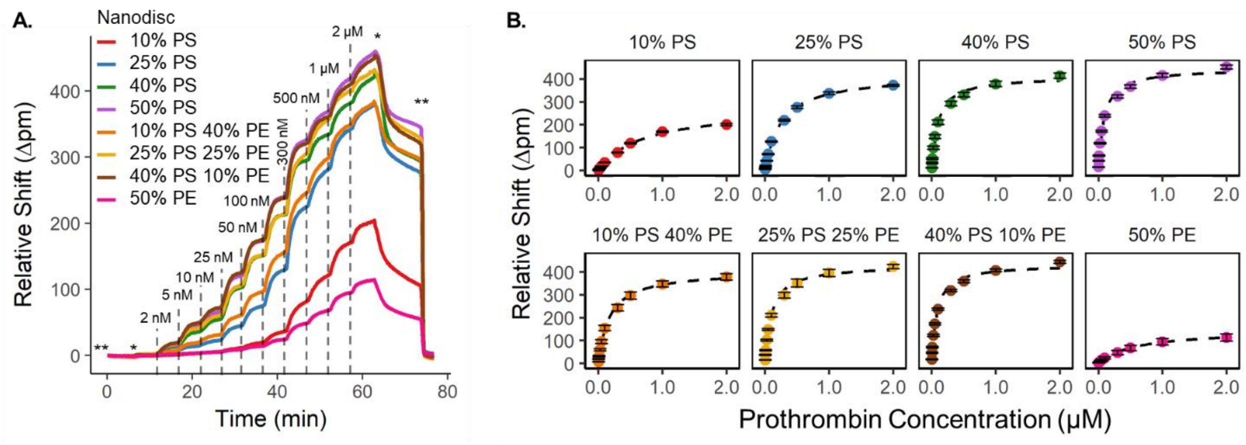

Figure S1: Prothrombin (PT) binding titration and K_d determination

For K_d determination, PT was flowed across a sensor chip, (Figure S1). The concentrations of PT used were 2 nM, 5 nM, 10 nM, 25 nM, 50 nM, 100 nM, 300 nM, 500 nM, 1000 nM, and 2000 nM. **A.** The PT binding response during a titration, flowed at 10 μL/min. Subtraction of binding to 100% PC Nanodiscs was used to correct for nonspecific binding. Each trace depicts the average binding response to a different Nanodisc: 10% PS (red), 10% PS 40% PE (orange), 25% PS (blue), 25% PS 25% PE (yellow), 40% PS (green), 40% PS 10% PS (brown), 50% PE (pink), and 50% PS (purple) all made with PC as the balance. The dashed lines indicate the addition of a new concentration of PT, the *marks the transition to HEPES buffer, and ** marks the transition to HEPES (-). **B.** The maximal shift of PT binding vs PT concentration fit to Equation (1) for each type of Nanodisc used.

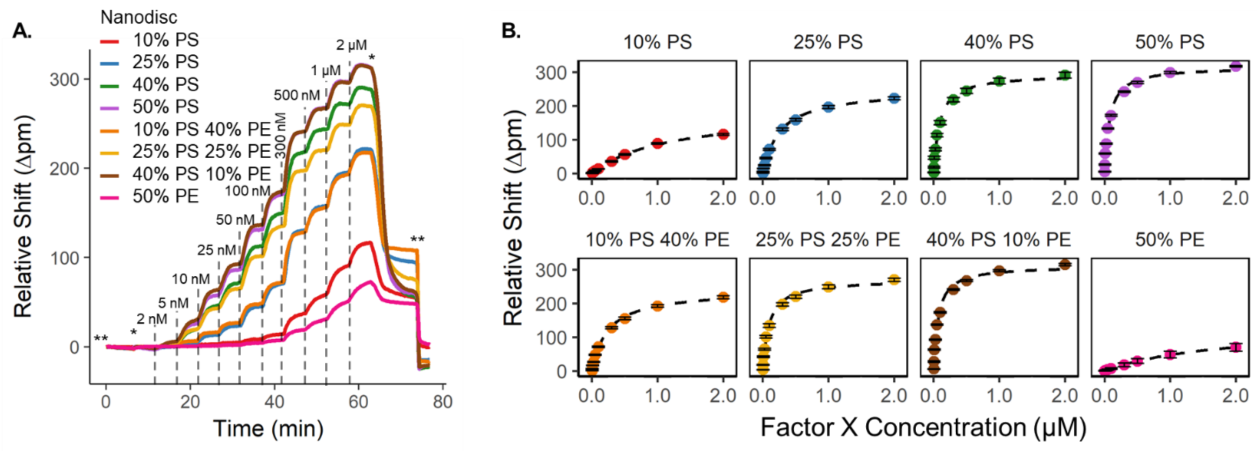

Figure S2: Factor X (fX) binding titration and K_d determination

For K_d determination, fX was flowed across a sensor chip, (Figure S1). The concentrations of fX used were 2 nM, 5 nM, 10 nM, 25 nM, 50 nM, 100 nM, 300 nM, 500 nM, 1000 nM, and 2000 nM. **A.** The fX binding response during a titration, flowed at 10 µL/min. Subtraction of binding to 100% PC Nanodiscs was used to correct for nonspecific binding. Each trace depicts the average binding response to a different Nanodisc: 10% PS (red), 10% PS 40% PE (orange), 25% PS (blue), 25% PS 25% PE (yellow), 40% PS (green), 40% PS 10% PS (brown), 50% PE (pink), and 50% PS (purple) all made with PC as the balance. The dashed lines indicate the addition of a new concentration of fX, the *marks the transition to HEPES buffer, and ** marks the transition to HEPES (-). **B.** The maximal shift of fX binding vs fX concentration fit to Equation (1) for each type of Nanodisc used.

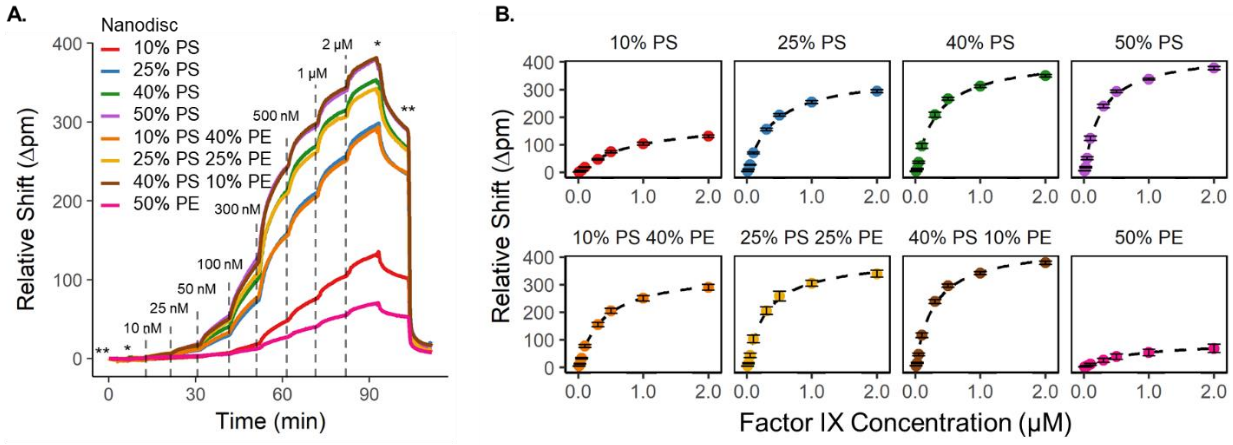

Figure S3: Factor IX (fIX) binding titration and K_d determination
For K_d determination, fIX was flowed across a sensor chip, (Figure S1). The concentrations of fIX used were 10 nM, 25 nM, 50 nM, 100 nM, 300 nM, 500 nM, 1000 nM, and 2000 nM. **A.** The fIX binding response during a titration, flowed at 10 µL/min. Subtraction of binding to 100% PC Nanodiscs was used to correct for nonspecific binding. Each trace depicts the average binding response to a different Nanodisc: 10% PS (red), 10% PS 40% PE (orange), 25% PS (blue), 25% PS 25% PE (yellow), 40% PS (green), 40% PS 10% PS (brown), 50% PE (pink), and 50% PS (purple) all made with PC as the balance. The dashed lines indicate the addition of a new concentration of fIX, the *marks the transition to HEPES buffer, and ** marks the transition to HEPES (-). **B.** The maximal shift of fIX binding vs fIX concentration fit to Equation (1) for each type of Nanodisc used.

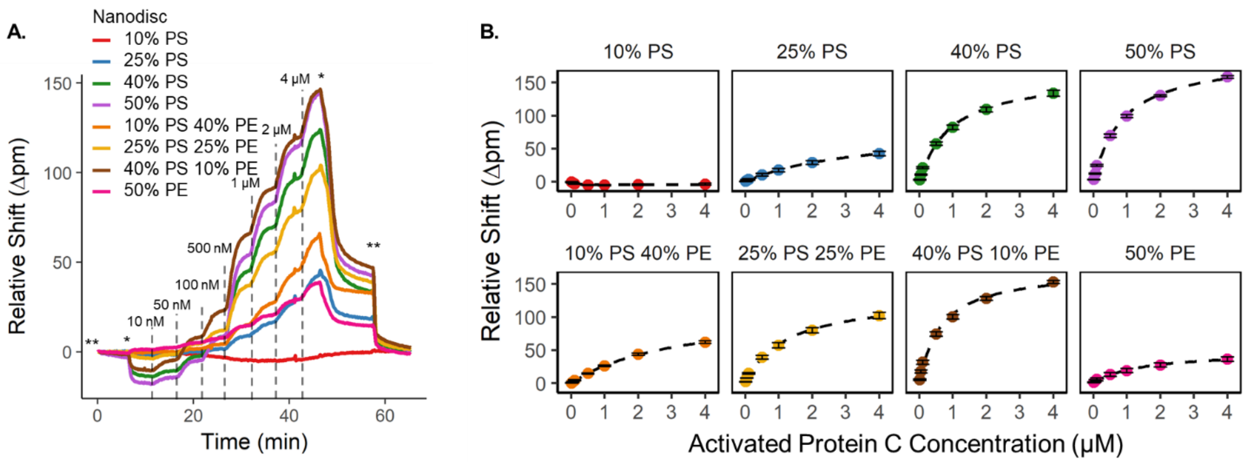

Figure S4: Activated protein C (aPC) binding titration and K_d determination

For K_d determination, aPC was flowed across a sensor chip, (Figure S1). The concentrations of aPC used were 10nM, 50 nM, 100 nM, 500 nM, 1000 nM, 2000 nM, and 4000 nM. **A.** The aPC binding response during a titration, flowed at 10 µL/min. Subtraction of binding to 100% PC Nanodiscs was used to correct for nonspecific binding. Each trace depicts the average binding response to a different Nanodisc: 10% PS (red), 10% PS 40% PE (orange), 25% PS (blue), 25% PS 25% PE (yellow), 40% PS (green), 40% PS 10% PS (brown), 50% PE (pink), and 50% PS (purple) all made with PC as the balance. The dashed lines indicate the addition of a new concentration of aPC, the *marks the transition to HEPES buffer, and ** marks the transition to HEPES (-). **B.** The maximal shift of aPC binding vs aPC concentration fit to Equation (1) for each type of Nanodisc used.

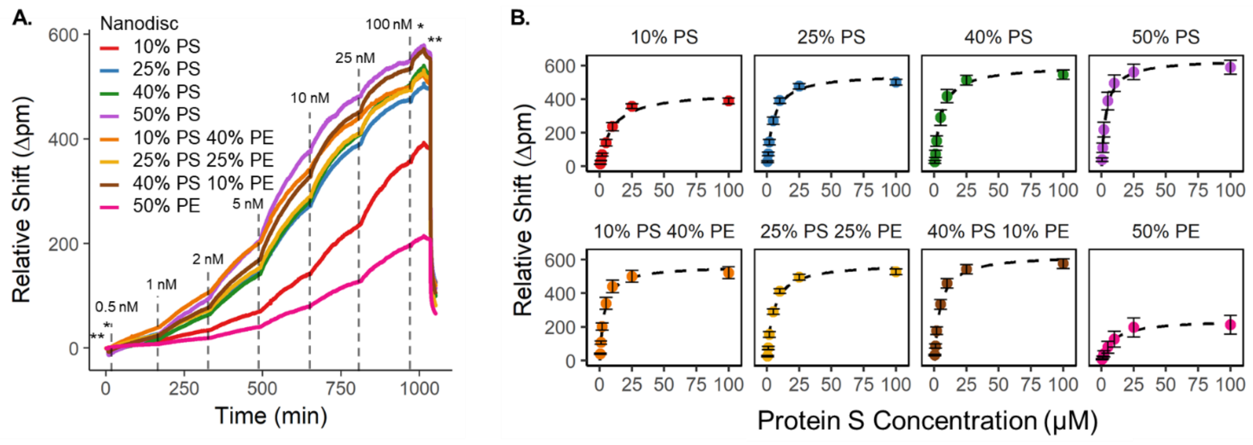

Figure S5: Protein S (PrS) binding titration and K_d determination

For K_d determination, PrS was flowed across a sensor chip, (Figure S1). The concentrations of PrS used were 10 nM, 25 nM, 50 nM, 100 nM, 300 nM, 500 nM, 1000 nM, 2000 nM, and 4000 nM. **A.** The PrS binding response during a titration, flowed at 10 µL/min. Subtraction of binding to 100% PC Nanodiscs was used to correct for nonspecific binding. Each trace depicts the average binding response to a different Nanodisc: 10% PS (red), 10% PS 40% PE (orange), 25% PS (blue), 25% PS 25% PE (yellow), 40% PS (green), 40% PS 10% PS (brown), 50% PE (pink), and 50% PS (purple) all made with PC as the balance. The dashed lines indicate the addition of a new concentration of PrS, the *marks the transition to HEPES buffer, and ** marks the transition to HEPES (-). **B.** The maximal shift of PrS binding vs PrS concentration fit to Equation (1) for each type of Nanodisc used.

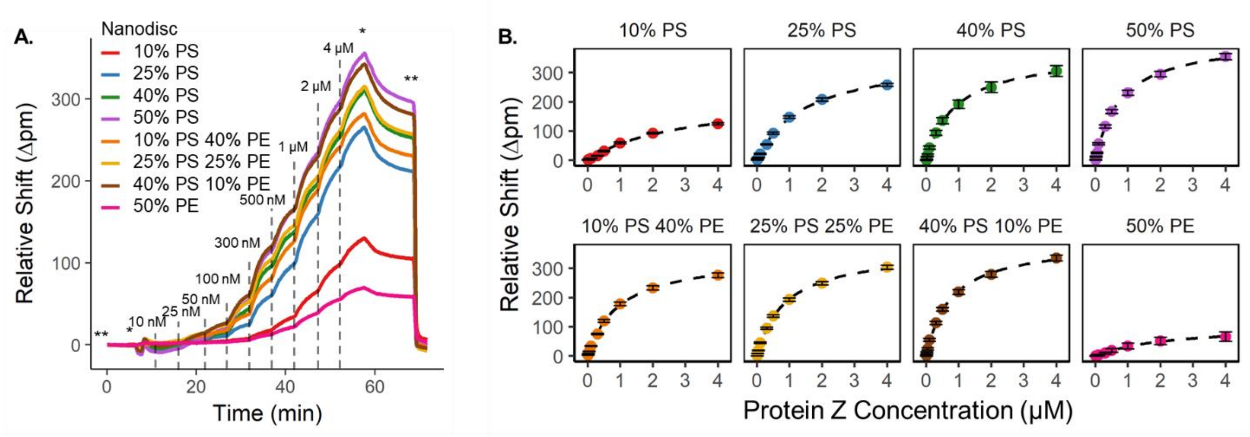

Figure S6: Protein Z (PrZ) binding titration and K_d determination

For K_d determination, PrZ was flowed across a sensor chip, (Figure S1). The concentrations of PrZ used were 10 nM, 25 nM, 50 nM, 100 nM, 300 nM, 500 nM, 1000 nM, 2000 nM, and 4000 nM. **A.** The PrZ binding response during a titration, flowed at 10 µL/min. Subtraction of binding to 100% PC Nanodiscs was used to correct for nonspecific binding. Each trace depicts the average binding response to a different Nanodisc: 10% PS (red), 10% PS 40% PE (orange), 25% PS (blue), 25% PS 25% PE (yellow), 40% PS (green), 40% PS 10% PS (brown), 50% PE (pink), and 50% PS (purple) all made with PC as the balance. The dashed lines indicate the addition of a new concentration of PrZ, the *marks the transition to HEPES buffer, and ** marks the transition to HEPES (-). **B.** The maximal shift of PrZ binding vs PrZ concentration fit to Equation (1) for each type of Nanodisc used.

Table S1: K_d values in nM of PT, fX, fIX, fVIIa, aPC, PrZ, and PrS

	10% PS	***25% PS***	***40% PS***	***50% PS***	***10% PS 40% PE***	***25% PS 25% PE***	***40% PS 10% PE***	***50% PE***
PT	670 ± 30	244 ± 8	93 ± 6	82 ± 1	190 ± 30	98 ± 2	76 ± 4	600 ± 200
fX	1130 ± 60	260 ± 10	87 ± 7	73 ± 2	250 ± 20	92 ± 5	67 ± 3	2000 ± 700
fIX	810 ± 30	390 ± 10	310 ± 30	260 ± 20	390 ± 30	290 ± 20	280 ± 20	800 ± 100
fVIIa	1800 ± 300	1260 ± 50	900 ± 90	790 ± 50	690 ± 50	740 ± 50	690 ± 50	860 ± 60
aPC	NA	3600 ± 1000	920 ± 80	880 ± 80	3200 ± 300	1210 ± 80	650 ± 80	1500 ± 600
PrS	9.7 ± 0.8	5.0 ± 0.5	5.3 ± 1.0	4.0 ± 0.3	3.8 ± 0.4	5.0 ± 0.4	4.7 ± 0.5	11 ± 2
PrZ	2800 ± 200	1430 ± 90	900 ± 60	780 ± 30	1010 ± 60	860 ± 30	750 ± 40	2500 ± 700

Error represents standard deviation from at least n=4 microrings in a single detection experiment.

NA values were unable to be calculated.
